# Morphological and Molecular Characterization of a New *Mycobacterium avium* Subsp. *paratuberculosis* S-Type Strain Genotype in Goats

**DOI:** 10.3389/fvets.2019.00250

**Published:** 2019-07-31

**Authors:** Simone Scherrer, Roger Stephan, Jon Paulin Zumthor, Anja Kipar, Frauke Seehusen

**Affiliations:** ^1^Section of Veterinary Bacteriology, University of Zurich, Zurich, Switzerland; ^2^Institute for Food Safety and Hygiene, University of Zurich, Zurich, Switzerland; ^3^Institute for Food Safety and Animal Health, Chur, Switzerland; ^4^Institute for Veterinary Pathology, University of Zurich, Zurich, Switzerland

**Keywords:** *Mycobacterium avium* subsp. paratuberculosis, goat, VNTR, S-type MAP, genotyping of tissue homogenates

## Abstract

Paratuberculosis is a chronic bacterial disease of global importance mainly in domestic and wild ruminants, caused by *Mycobacterium avium* subsp. *paratuberculosis* (MAP). In goats, paratuberculosis is mostly caused by the “C-type” (cattle) and in a few cases by the “S-type” (sheep) strain of MAP. In 2017, a caprine S-type III isolate with a new VNTR profile was identified in a Swiss alpine region. In 2018, new caprine isolates with the same novel VNTR profile originating from a farm of a close by neighboring valley were analyzed. Here we report on this MAP S-type III outbreak in a Swiss dairy goat farm in which we investigated the pathological changes, distribution and genotype of MAP tissue homogenates. Full necropsy and histological examination were undertaken on two female adult goats with a history of weight loss and intermitting diarrhea. Routine and special stains were applied to characterize the morphological changes. DNA was extracted from 33 different tissue samples and tested for MAP by qPCR targeting IS*900* and F57. Subtyping was performed, using the variable number tandem repeats (VNTR) and mycobacterial interspersed repetitive units (MIRU) approach. The goats showed moderate to marked emaciation and displayed typical clinical features of paratuberculosis. A moderate granulomatous enteritis and regional lymphadenitis with a small to moderate number of acid-fast bacteria within macrophages was detected. MAP detection was mainly restricted to the gastrointestinal tract, mesenteric and hepatic lymph nodes. Subtyping the S-type isolates using a panel of eight established MIRU-VNTR loci identified a new genotype, INMV 218.

## Introduction

Paratuberculosis or Johne's disease is characterized by a chronic progressive granulomatous enteritis caused by the pathogen *Mycobacterium avium* subsp. *paratuberculosis* (MAP). The disease is distributed worldwide in the bovine population, in small ruminants such as goats and sheep, as well as in other ruminant species (1). The usual route of MAP infection is fecal-oral, with young cattle primarily becoming infected by exposure to feces from infected adult cattle or their environment. MAP is introduced in ruminant herds through trading of subclinically infected animals (2), although wildlife reservoirs might also play a role in MAP transmission to livestock (3).

Based on its association with host species, MAP has been allocated to two major groups, the “Sheep-type” (also called “S-type” or “type I and III”) and the “Cattle-type” (also called “C-type” or “type II”) (4, 5). More commonly, goats with paratuberculosis are infected by C-type MAP, however, S-type infections of goats are not rare and have been described previously (6). S-type strains can be further subdivided into type I and the closely related type III (intermediate type), which show a wider host range (7, 8). In contrast to the C-type strains, S-type MAP grow much slower and more fastidious in culture media (9, 10). In general, C-type MAP has a higher infection ability and better surviving capacity in macrophages compared to S-type MAP (11).

In 2017 a Swiss S-type strain of a caprine isolate was identified with a new VNTR profile. In 2018, new caprine isolates from the same alpine region of a farm originating from a close by neighboring valley were found to have an identical VNTR profile. Therefore, the question was raised if this strain causes a different epidemiological or pathological pattern. Consequently, a study was designed to address the pathological changes, distribution and genotype of MAP found in tissue and fecal samples of goats originating from this dairy goat farm.

## Materials and Methods

### Animals and Samples

2 female adult goats from a farm in the canton of Grisons with a clinical history of weight loss and intermitting diarrhea were submitted for a post mortem examination upon the owner's request. Both were euthanized in order to determine the cause of illness with respect to herd health. Gross examination was carried out and samples collected from various tissues for histological examination and DNA extraction were analyzed (Table 1). In order to monitor the prevalence of MAP within the affected farm, PCR of fecal samples of the whole livestock was screened for MAP. Twenty-four caprine fecal samples and two bulk fecal samples of the remaining livestock comprising 27 cows and 40 sheep, respectively, were examined. Moreover, a caprine isolate of a diagnostic submission to the Swiss section of Veterinary Bacteriology Laboratories in 2017, originating from a farm of a close by neighboring valley, was analyzed by VNTR as well (Data not shown).

**Table 1:** Distribution of MAP in tissues, the corresponding VNTR code and obtained histological lesion scores of two tested goats are represented.

	**Goat 1**											**Goat 2**										
	**in-house qPCR**				**VNTR code^a^**	**qPCR^b^**				**qPCR^c^**	**Histological lesion scores^d^**	**in-house qPCR**				**VNTR code^a^**	**qPCR^b^**				**qPCR^c^**	**Histological lesion scores^d^**
**Tissue Sample**	**C_**T**_ F57**	**C_**T**_ IS*900***	**C_**T**_ IAC**	**Result**	**292|X3|25|47|3|7|10|32**	**C_**T**_ IS900**	**pg/μl**	**Bacteria/ mg tissue**	**Result**	**Result**		**C_**T**_ F57**	**C_**T**_ IS*900***	**C_**T**_ IAC**	**Result**	**292|X3|25|47|3|7|10|32**	**C_**T**_ IS900**	**pg/μl**	**Bacteria/mg tissue**	**Result**	**Result**	
Duodenum	–	37.9	33.1	–	n.p.	31.75	0.0002	1	–	–	Diffuse lympho(plasma)cytic/ type IIIc; no AFB	–	39.5	32.6	–	n.p.	32.62	0.0001	0	–	–	n.p.
Jejunum including Jejunal Peyer's patches (JPP)	25	23.2	34.9	+	4-2-3-3-1-1.5-1-8	17.2	6.6976	20,093	+	+	Diffuse mixed/type IIIb/diffuse intermediate; moderate number of AFB	–	39.5	32.8	–	n.p.	31.67	0.0007	2	–	–	Diffuse lympho(plasma)cytic/type IIIc; small numbers of AFB
Ileum + Ileal Peyer's patches (IPP)	23	20.8	33.4	+	4-2-3-3-1-1.5-1-8	14.99	33.3127	99,938	+	+	Diffuse mixed/type IIIb/diffuses intermediate; moderate number of AFB	30.7	29.1	32.2	+	4-2-3-3-1-1.5-1-8	22.75	0.2777	833	+	+	Diffuse mixed/type IIIb/diffuse intermediate; moderate numbers of AFB
Ileocaecal valve (ICVPP)	23	20.2	32.9	+	4-2-3-3-1-1.5-1-8	14.8	38.3108	114,932	+	+	n.p.	29	26.9	33.3	+	4-2-3-3-1-1.5-1-8	20.57	1.1903	3,571	+	+	n.p.
Caecum	25	23.2	33.5	+	4-2-3-3-1-1.5-1-8	16.67	9.8713	29,614	+	+	Diffuse lympho(plasma)cytic /type IIIc, single AFB	32	30.1	34.1	+	4-2-3-3-1-1.5-1-8	23.22	0.2027	608	+	+	Diffuse lympho(plasma)cytic/type IIIc; no AFB
Colon ascendens	26	24.3	33	+	4-2-3-3-1-1.5-1-8	17.47	5.5109	16,533	+	+	Diffuse lympho(plasma)cytic /type IIIc, single AFB	33.1	32	32.9	+	4-2-3-3-1-1.5-n.a.-n.a.	25.6	0.0415	125	+	+	Diffuse lympho(plasma)cytic/type IIIc; no AFB
Colon transversum	29	26.4	33	+	4-2-3-3-1-1.5-1-8	20.16	0.7817	2,345	+	+	Diffuse lympho(plasma)cytic /type IIIc, single AFB	–	37.4	33.3	–	n.a.-2-3-n.a.-n.a.-n.a.-n.a.-n.a.	28.91	0.0046	14	+	–	Diffuse lympho(plasma)cytic/type IIIc; no AFB
Colon descendens	31	29.1	33.1	+	n.a.-2-3-3-1-1.5-1-8	22.43	0.1516	455	+	+	Diffuse lympho(plasma)cytic /type IIIc, single AFB	36.3	37.1	32.6	+	n.a.-n.a.-n.a.-n.a.-1-n.a.-n.a.-n.a.	28.24	0.0071	21	+	+	Diffuse lympho(plasma)cytic/type IIIc; no AFB
Rectum	29	27.1	32.6	+	4-2-3-3-1-1.5-1-8	20.3	0.708	2,124	+	+	n.p.	-	36.6	33.6	–	n.p.	30.06	0.0021	6	+	+	n.p.
Rumen	37	33.6	33.7	+	4-2-3-3-1-n.a.-1-8	26.64	0.0071	21	+	+	n.p.	36.2	34.4	33.6	+	n.a.-n.a.-n.a.-n.a.-1-1.5-n.a.-n.a.	26.94	0.017	51	+	+	n.p.
Abomasum	-	37.7	33	–	n.p.	28.79	0.0015	5	+	+	n.p.	35.4	33.5	32.4	+	n.p.	26.97	0.0167	50	+	+	n.p.
Colonic lymph node (Co-LN)	38	37.3	33.4	+	n.a.-2-3-n.a.-1-1.5-1-8	28.85	0.0014	4	+	–	n.p.	36.4	34.2	33.6	+	4-2-3-3-1-1.5-1-n.a.	27.11	0.0152	46	+	+	n.p.
Mesenteric lymph node (M-LN)	31	28.6	33.3	+	4-2-3-3-1-1.5-1-8	22	0.2065	620	+	+	n.p.	37.9	35.7	33.3	+	n.a.-2-3-n.a.-n.a.-n.a.-1-n.a.	29.37	0.0034	10	+	+	n.p.
Ileocaecal valve lymph node (ICV-LN)	35	32.8	32.7	+	4-2-3-3-1-1.5-1-8	25.99	0.0114	34	+	+	n.p.	34.7	33.1	32.3	+	4-2-3-3-1-1.5-1-8	25.73	0.0381	114	+	+	n.p.
Hepatic lymph node	33	30.5	34.2	+	4-2-3-3-1-1.5-1-8	24.42	0.0358	107	+	+	n.p.	–	–	33.5	–	n.p.	29.85	0.0011	3	+	–	n.p.
Superficial cervical lymph node	–	37.8	33.9	–	n.p.	31.5	0.0002	1	–	–	n.p.	–	–	32.7	–	n.p.	31	0.0005	2	–	–	n.p.
Superficial inguinal lymph node	–	35.9	33.3	–	n.p.	29.87	0.0007	2	–	–	n.p.	–	–	34.1	–	n.p.	30.91	0.0005	2	–	–	n.p.
Mandibular lymph node	–	35.4	32.6	–	n.p	29.32	0.001	3	+	–	n.p.	–	–	33.8	–	n.p.	30.82	0.0005	2	–	–	n.p.
Tonsils	–	–	34.1	–	n.p.	31.77	0.0002	1	–	–	n.p.	–	–	33.1	–	n.p.	31.89	0.0003	1	–	–	n.p.
Spleen	–	–	32.9	–	n.p.	30.17	0.0006	2	–	–	n.p.	–	–	33.9	–	n.p.	30.24	0.0008	2	–	–	n.p.
Kidney	–	–	34.1	–	n.p.	31.48	0.0002	1	–	–	n.p.	–	–	33.6	–	n.p.	30.68	0.0006	2	–	–	n.p.
Liver	–	35.1	33.6	–	n.p.	28.34	0.0021	6	+	+	n.p.	–	38.5	33.3	–	n.p.	31.4	0.0004	1	–	–	n.p.
Diaphragm	–	43.9	34.3	–	n.p.	30.25	0.0005	2	-	-	n.p.	–	39.6	33	–	n.p.	29.52	0.0013	4	+	+	n.p.
Gluteal muscle	–	35.6	33.5	–	n.p.	29.54	0.0009	3	–	–	n.p.	–	–	33.3	–	n.p.	28.63	0.0025	8	+	–	n.p.
Lung	–	41	33.1	–	n.p.	31.02	0.0003	1	–	–	n.p.	–	–	33.3	–	n.p.	29.72	0.0002	1	–	–	n.p.
Heart	–	–	33.8	–	n.p.	31.06	0.0003	1	–	–	n.p.	–	–	32.8	–	n.p.	31.17	0.0004	1	–	–	n.p.
Aorta	29	26.8	33	+	4-2-3-3-1-1.5-n.a.-8	19.84	0.9859	2,958	+	+	n.p.	–	–	33	–	n.p.	29.21	0.0037	11	+	+	n.p.
Pancreas	–	–	32.6	–	n.p.	31.7	0.0002	1	–	–	n.p.	–	–	34.3	–	n.p.	31.69	0.0003	1	–	–	n.p.
Adrenal	–	–	34.1	–	n.p.	31.84	0.0002	1	–	–	n.p.	–	–	34	–	n.p.	32.9	0.0001	0	–	–	n.p.
Bone marrow	–	36.3	33.3	–	n.p.	28.48	0.0019	6	+	+	n.p.	–	–	33	–	n.p.	29.81	0.0011	3	+	–	n.p.
Udder	–	37.8	32.8	–	n.p.	30.21	0.0005	2	–	–	n.p.	–	37.4	33.1	–	n.p.	29.93	0.001	3	+	–	n.p.
Salivary gland	–	–	32.8	–	n.p.	30.76	0.0004	1	–	–	n.p.	–	39.8	32.6	–	n.p.	28.87	0.0021	6	+	–	n.p.
Brain	–	–	34.7	–	n.p.	29.92	0.0007	2	–	–	n.p.	–	37.4	33	–	n.p.	31.72	0.0003	1	–	–	n.p.

*Results of an in-house developed qPCR assay are represented in comparison to the published and extensively validated qPCR by Plain et al. (12) and a commercially available qPCR kit*.

a *MIRU-VNTR analysis according to Thibault et al. (13)*.

b *qPCR according to Plain et al. (12) the cut point ≥ 0.001 pg/MAP DNA was applied to calculate Bacteria/mg tissue: 10 fg DNA corresponded to 30 MAP Bacteria with a estimated IS900 copy number of 15*.

c *Results of a commercially available Kit (LSI VetMax Triplex MAP, Thermo Scientific)*.

d *Tissue lesion grade identified by histolopathological examination according to Pérez et al. (14), Corpa et al. (15), Balseiro et al. (16)*.

*n.p.: VNTR analysis/histopathology not performed*.

*n.a.: no amplification of PCR reaction*.

*AFB: acid fast bacteria*.

In accordance with local legislation, ethical approval was not required and no animal experiments were carried out for this study.

### Histological and Immunohistological Examination

Tissue samples were fixed in 4% buffered formalin and routinely embedded in paraffin wax. Sections (3–5 μm) were prepared and routinely stained with hematoxylineosin (HE). For samples from the small and large intestine as well as mesenteric lymph nodes, consecutive sections were stained with Ziehl Neelsen stain for the detection of acid fast bacteria. In small intestine and lymph node tissue, an immunohistochemical examination using antibodies directed against macrophages (Iba1; Wako Chemicals, Neuss, Germany, Ref. 019-19741, dilution 1:750) as well as T lymphocytes (CD3; Dako, Agilent Technologies, Glostrup, Denmark, clone F7.2.38, Ref. M725401, dilution 1:150) and B lymphocytes (CD79a, Bio-Rad, clone HM57, Ref. MCA2538T, dilution 1:3000) was performed as previously described (17). Briefly, after deparaffinization and rehydration, samples were properly subjected to the adequate antigen retrieval treatment (CD3 and CD79a: heat, pH buffer 9.0; Iba1: heat, pH buffer 6.0). As secondary antibodies anti-mouse (CD3 and CD79a) and anti-rabbit (Iba1) EnVision HRP system (code no. K4001/4003, Dako, Agilent Technologies, Glostrup, Denmark) were applied. Furthermore, visualization was achieved by diaminobenzidine. Finally, slides were slightly counterstained with hemalaun and mounted. The histological scores of intestinal lesions were performed as described previously (14–16).

### DNA Extraction From Fecal Material and Tissue Samples

Tissue samples for DNA extraction were frozen at −20°C. For DNA extraction, a small piece of tissue (about 500 mg−1 g) was cut off the thawed samples and incubated in 360 μl ATL buffer (Qiagen, Hilden, Germany). DNA of fecal samples was extracted by adding 1 ml ATL buffer (Qiagen) to 0.5 g of homogenized stool sample. Subsequently, samples were transferred onto a bead beating matrix in a 2-ml microtube (Omni International, Kennesaw, USA), heat inactivated at 99°C and subjected to mechanical cell lysis (TissueLyser II, Qiagen) followed by enzymatic digestion with 40 μl proteinase K (Qiagen). Automated DNA preparation using 200 μl digested sample was performed on the QIAcube instrument using the QIAamp cador Pathogen Mini Kit (Qiagen) following the manufacturer's recommendation with a DNA elution volume of 100 μl. The obtained volume of eluted DNA corresponded to ~ 500 mg tissue. DNA concentration was measured using a NanoDrop 2000c Spectrophotometer (Thermo Scientific, Reinach, Switzerland) and the extracts stored at −20°C until use. Handling of samples followed standard biosecurity protocols.

### qPCR

All samples were examined for the presence of the F57 gene and the repetitive element IS*900* of MAP using a newly developed triplex qPCR. A probe (F57ss_probe) targeting the F57 gene (GenBank accession number: X70277.1) was designed with the sequence FAM-CTGGACCGCCGCTGACGCAC-BHQ1 and used at a final concentration of 100 nM. 400 nM forward primer F57ss_for (TTCCCGTCGATGACAGCG) was combined with 400 nM reverse primer MAPf57p2 (AGTGGGAGGCGTACCA) published previously (18). To target IS*900* (GenBank accession number: AJ250018.1), 100 nM of probe IS900ss_probe (YakkimaYellow-AGAGCCGTGCCGCGCTGATC-BHQ-1) was combined with 400 nM primer pair IS900ss_for (GGAACGCGCCTTCGACTA) and IS900ss_rev (CAAGAACGCGGCTACTCGA). An exogenous internal amplification control (IAC) was introduced for monitoring each reaction, since DNA preparations originating from fecal or tissue samples can contain inhibitory substances. Five femtogram (fg) of the pEGFP-1 standard vector (BD Bioscience Clonetech, USA) was used as IAC template, and a 177 bp amplicon (19) was amplified using 200 nM primer pair eGFP-1-F (GACCACTACCAGCAGAACAC) and eGFP-10-R (GAACTCCAGCAGGACCATG) and 25 nM of probe (ATTO 647 N**-**AGCACCCAGTCCGCCCTGAGCA**-**BHQ-3**)**. The PCR was performed on an ABI7500 Instrument (Thermo Scientific) using the Path-ID^TM^ qPCR Master Mix (Thermo Scientific). The qPCR reactions were performed in a total volume of 10 μl with 1 μl of five-fold diluted template DNA extracts. The cycling conditions were 10 min at 95°C, followed by a two-step cycling stage of 45 cycles of 15 s at 95°C and 60 s at 61°C.

In order to find the limit (LOD) of detection of the developed in-house qPCR, triplicates of a 10-fold series of dilution of genomic DNA of MAP reference strain ATCC 19698, ranging from 100 to 0.001 pg/μl corresponding to known concentrations of genome equivalents (GE), were measured. With a whole genome size of 4.83 Mb, a quantity of 5.3 fg was calculated for one GE of MAP. The LOD was determined as lowest dilution with amplification of all triplicates having a threshold cycle (C_T_) of C_T_ ≤ 38 and a standard deviation of ≤ 0.5. Samples were considered positive, if both target genes, F57 and IS*900*, were detected. With the purpose of specificity testing of the developed in-house qPCR an exclusivity panel of 30 *Mycobacteria* spp. was tested (Supplementary Table 1).

In order to validate the newly developed in-house qPCR, samples were verified with a well characterized and validated qPCR assay developed by Kawaji et al. (20) and Plain et al. (12), which is successfully applied in Australia for detection of MAP infection in suspected animals and flocks. This qPCR for IS*900* was performed in a total volume of 15 μl comprising 7.5 μl of SensiFASTSYBR No-ROX qPCR mastermix (Bioline), 1 μl of five-fold diluted template DNA extracts and 400 nM final concentration of each forward [MP10-1 (5′-ATGCGCCACGACTTGCAGCCT-3′)] and reverse primer [MP11-1 (5′-GGCACGGCTCTTGTTGTAGTCG-3′)] primers (20). The qPCR assay was carried out on the Rotor-Gene Q instrument (Qiagen) with the following thermocycling conditions: initial denaturation at 95°C for 3 min, 40 cycles at 95°C for 5 s and annealing/extension at 68°C for 30 s followed by a second cycling step at 95°C for 5 s and 40°C for 2 min. Finally, a high resolution melting analysis from 80 to 95°C was performed. Fluorescence data were acquired at 0.1°C increments every 2 s to generate specific melting curves. For quantifying MAP DNA, a 10-fold dilution series of genomic DNA of MAP reference strain ATCC19698, ranging from 100 to 0.001 pg/μl, was added to each set of sample analysis. C_T_ values were converted into quantitative results by using the obtained standard curve of each experimental set as a base for the calculation of the appropriate amount of MAP pg/μl (corresponding to pg MAP DNA/mg tissue). As suggested by Plain et al. (12) a DNA quantity cut point (≥0.001 pg MAP DNA) was applied. Resulting MAP-specific melting curves were verified to lie within a melting temperature (*T*_*m*_) range of 89.1 ± 0.1.

For further confirmation of the obtained qPCR results a commercially available probe-based kit for detection of IS*900* and F57 kit (LSI VetMax Triplex MAP, Thermo Scientific) was used according to the manufacturer's instructions.

### Genotyping by MIRU-VNTR

DNA preparations from F57 gene and IS*900* PCR positive samples were analyzed using eight established MIRU-VNTR targets (13). Each reaction mixture contained 1 × HotStarTaq Master Mix (Qiagen), 1 × Q-Solution (Qiagen) for loci VNTR 10 and VNTR 32 (no additional Q-buffer for VNTR loci 292, X3, 25, 47, 3, and 7), 0.5 μM of each primer pair and 1 ng purified mycobacterial DNA from PCR-positive samples in a final volume of 10 μl. PCR was performed with an initial 15 min activation step at 95°C, followed by 45 cycles of 95°C for 30 s, 60°C for 30 s, 72°C for 30 s, and a final extension step of 72°C for 10 min. Ten microliter of each PCR amplification product was analyzed using a capillary electrophoresis device QIAxcel (Qiagen), applying the OH1700 AM10sec method with a QX DNA high-resolution cartridge, QX 15 bp−3 kb alignment marker and QX 100 bp−2.5 kb size marker. Peak size assignment and allele code exportation was performed with the QIAxcel ScreenGel Software version 1.3.0 (Qiagen). As a positive control, the reference strain MAP ATCC 19698 was analyzed for each PCR run. For each MIRU-VNTR locus, the number of tandem repeats was determined from the size of the amplicons. Repeat numbers of MIRU-VNTR loci were assigned according to a previously described allele-calling table and compared to profiles called INMV (INRA Nouzilly MIRU-VNTR). The new profile detected was registered in the MAC-INMV database (http://mac-inmv.tours.inra.fr/).

The differentiation between C-type and S-type MAP was determined as described by Leao et al. (21). Briefly, the differentiation between C-type and S-type and S-type I and III, respectively, was determined by a single nucleotide polymorphism (SNP) assay based on PCR and restriction enzyme digestion of amplified PCR products. In a final volume of 20 μl, snp3842359 was amplified using 0.5 μM of each primer pair MAP_F1 and MAP_R1 or primer pair TypeS_F and TypeS_R, respectively, for snp343677 using 2x HotStarTaq Master Mix (Qiagen) and 1 μl 5-fold diluted DNA template. PCR cycling conditions were as follows: initial 15 min activation step at 95°C, followed by 45 cycles of 95°C for 30 s, 60°C for 30 s, 72°C for 30 s, and a final extension step of 72°C for 10 min. Nine microliter of the obtained PCR products comprising the respective SNPs were digested using *BsmB* I and *Ava* II (Thermo Scientific, Schlieren, Switzerland), respectively, according to the manufacturer's instructions. Restricted products were visualized using the capillary electrophoresis device QIAxcel (Qiagen) by applying a QIAxcel DNA screening gel cartridge, QX 15 bp−3 kb alignment marker and QX 100 bp−2.5 kb size marker. The size analysis of the corresponding restriction fragments resulted in the discrimination of MAP into C-type or S-type and S-type I or III, respectively. For further confirmation of S-type classification sequence analysis of SNPs within *gyr*A and *gyr*B according to Biet et al. was performed (22) (Data not shown).

## Results

### Pathological Findings

The gross examination revealed that goat 1 was severely emaciated, with total loss of adipose tissue and serous atrophy of bone marrow (consistent with cachexia), whereas goat 2 was mildly to moderately emaciated. In both animals, mesenteric, ileocolic, and colonic lymph nodes were moderately to severely enlarged and showed a bulging cut surface, without evidence of necrosis or calcification. The intestine (aboral part of the small intestine) exhibited mild to moderate diffuse thickening of the mucosa.

**Figure 1:**
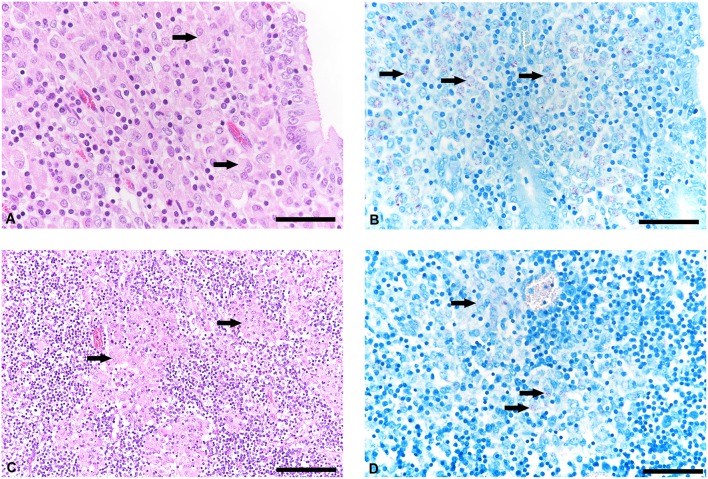
Histological lesions in jejunum and mesenteric lymph node and detetction of acid-fast bacteria in tissue samples of goats with paratuberculosis (goat 1). **(A)** Infiltration of the jejunal lamina propria with predominantly macrophages (arrows), hematoxylineosin (HE) stain, bar = 50 μm. **(B)** Small to moderate number of acid-fast intrahistiocytic bacteria (arrows), Ziehl Neelsen stain, bar = 50 μm. **(C)** Mesenteric lymph node with granulomatous inflammation (arrows), HE stain, bar = 100 μm. **(D)** Detection of acid-fast bacteria in the cytoplasm of macrophages (arrows), Ziehl Neelsen stain, bar = 50 μm.

The histological examination revealed a moderate granulomatous enteritis affecting the jejunum and ileum in goat 1 and the ileum in goat 2. This was represented by a diffuse infiltration of the lamina propria with macrophages with often abundant eosinophilic, partly granulated to foamy cytoplasm (epitheloid macrophages; Figure 1A). Multinucleated cells were rarely detected. The Ziehl Neelsen stain revealed low to moderate numbers of acid fast bacilli within the cytoplasm of the epitheloid macrophages (Figure 1B). In the large intestine, changes were restricted to a mild to moderate diffuse infiltration of the lamina propria with lymphocytes, plasma cells and non-epitheloid macrophages, consistent with a mild to moderate chronic lymphoplasmacellular typhlocolitis. Only in a few areas, a small number of epitheloid macrophages with single cytoplasmic acid-fast bacteria was detected. The histologically investigated mesenteric lymph nodes were also infiltrated by the epitheloid macrophages (Figure 1C). The Ziehl Neelsen stain also revealed acid fast bacilli of the same amount as in the small intestine within the cytoplasm of macrophages (Figure 1D). In general, the severity of the lesions and the number of bacteria were reduced in goat 2 compared to goat 1. In the lamina propria of the small intestine, there was a mixture of Iba1-positive macrophages (mainly in the villi) and CD3-positive lymphocytes (in villi and crypts as well as intraepithelial). CD79a-positive B cells were present in small numbers mainly at the base of the crypts but also in the villi.

The histological scores of intestinal lesions are illustrated in Table 1. Jejunum and ileum in goat 1 and ileum in goat 2 displayed the so called diffuse mixed/diffuse intermediate type, also named type IIIb. Additionally, moderate numbers of intrahistiocytic acid fast bacteria were detected in these lesions. Furthermore, other segments of small and large intestines displayed a diffuse lympho(plasma)cytic/type IIIc with small or single numbers of acid fast bacteria. Especially in goat 2, mild inflammatory lesions in the large intestine did not show any acid fast bacilli.

The lymph nodes in both animals showed a mild follicular hyperplasia with elevated numbers of CD79a-positive B cells in the follicular center and moderately elevated numbers of CD3-positive T cells in the paracortical region displaying a moderate paracortical lymphoid hyperplasia. Iba1-positive macrophages were mainly found in the medullar region of the lymph node but also as nodular accumulations with an epitheloid morphology in the perifollicular area (Supplementary Figure S1).

In addition, the lung of goat 2 displayed multiple small (5 to 20 mm in diameter) nodules that were histologically represented by moderate multifocal infiltrates of macrophages (often with an epitheloid appearance) and multinucleated giant cells as well as lymphocytes, plasma cells and individual eosinophilic granulocytes surrounding a central necrotic area with embedded remnants of nematodes. There were no further gross findings, and the histological examination did not reveal any pathological changes in any organ (including the aortic tissue of goat 1 which was examined histologically due to a positive PCR result) apart from the myocardium of both animals, where multiple protozoan cysts consistent with *Sarcocystis* sp. were found randomly distributed without any tissue or inflammatory reaction.

### qPCR

The specificity of the developed in-house qPCR was tested by performing an exclusivity panel of 30 mycobacterial DNA according to Supplementary Table 1. None of the strains showed any signal for F57. For IS*900* as target, however, the following 7 strains gave rise to C_T_ values between 36.3 and 37.5: *M. avium* subsp. *silvaticm, M. scrofulaceum, M. simiae, M. parafortuitum, M. malmoense, M. xenopi*, and *M. abscessus*. Therefore, only results with positive C_T_ values for both targets F57 and IS*900* where considered positive.

The LOD for the lowest dilution for which the acceptance criteria (standard deviation ≤ 0.5 and C_T_ value ≤ 38) were fulfilled was 10 GE for F57 and 1 GE for IS*900* (corresponding to 15 MAP bacteria calculated with a copy number of 15 IS*900* insertion elements).

The in-house qPCR assay provided a system for MAP identification in samples with a cut-off value of 10 GE, which allowed to detect MAP from tissue samples in agreement with a commercially available kit and a published qPCR assay (12, 20) with an exception of samples below the threshold level. C_T_ values higher than 35 for IS*900* and C_T_ values higher than 38 for F57 are expected to reflect background contamination due to a bacterial content of <10 bacteria per sample tested.

In contrast, a detection level of 1 fg MAP DNA corresponding to approximately one-fifth of a MAP genome [corresponding to 3–4 bacteria with a multicopy presence of insertion element IS*900* of 15 to 20 copies per genome (23)] was successfully obtained when performing the extensively validated qPCR assay of Plain et al. (12) and therefore giving rise to additional positive results with low numbers of bacteria (<10).

### Identification of MAP and Genotyping by MIRU-VNTR

**Figure 2:**
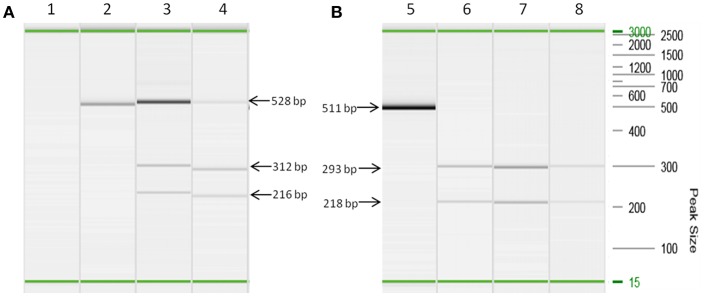
Strain typing of directly isolated DNA deriving from tissue and fecal samples based on a PCR-restriction endonuclease analysis genotyping assay (21). **(A)** Lanes 2, 3, and 4 are PCR amplicons of snp3842359 (for C-type and S-type MAP differentiation) from DNA extracts of lymph node tissues (lane 3) and fecal sample (lane 4) restricted with *BsmB* I. Lane 2 corresponds to culture isolate MAP ATCC 19698, which remains undigested by *BsmB* I corresponding to the pattern of a C-type genotype. Lane 1 is a negative control. **(B)** Lanes 5, 6, 7, and 8 are PCR amplicons of snp343677 (for S-type I and III differentiation) from DNA extracts of lymph node tissues (lane 6), intestine tissues (lane 7) and fecal sample (lane 8) restricted with *Ava* II. Lane 5 is the undigested PCR amplicon of a S-type MAP strain. All PCR reactions were performed using prediluted DNA extracts (1:5 dilution) in order to prevent inhibition of PCR. On the right hand side the DNA size marker (100 bp−2.5 kb) is represented. In each capillary electrophoresis run an alignment marker of 15 bp and 3,000 kb was loaded representing the start and end of electrophoresis. Visible minor differences of apparent size of PCR products are artifacts because PCR reactions have been carried out at different days. Therefore, this figure illustrates a merged picture of several capillary electrophoresis runs. Fragment sizes of PCR products and restriction products, respectively, indicated by arrows apply to all corresponding visible bands.

Both MAP F57 and IS*900* were successfully amplified in considerable amounts from ileum, caecum, colon (mostly affected colon ascendens) and rumen for both animals goat 1 and 2. Jejunum, rectum and aorta were only affected in goat 1, whereas samples from rectum and aorta were still detectable around the threshold level of 10 bacteria when testing the samples with the qPCR of Plain et al. (12) and with the commercial qPCR. When analyzing the lymph node tissues, goat 1 showed a positive signal mainly for the mesenteric and hepatic lymph nodes and to a lower extent for the ileocaecal valve lymph node while on the other hand goat 2 was positive for colonic and ileocaecal valve lymph nodes and mesenteric lymph node only around the threshold level. Both animals showed the highest amount of MAP in ileum and ileocaecal valve (Table 1). The examination of the caprine fecal samples originating from the livestock of the concerned farm yielded in only two low level shedding animals. The bovine and ovine bulk samples were PCR negative for MAP. The caprine isolate of 2017 from the close by neighboring valley was MAP PCR-positive. All MAP PCR-positive samples were subtyped and confirmed as S-type III by PCR-restriction endonuclease analysis using directly extracted DNA from tissue or fecal samples (Figure 2) and sequence analysis of *gyr*A and *gyr*B. The MIRU-VNTR genotyping identified the same novel genotype in intestinal samples of rectum, rumen, as well as mesenteric and hepatic lymph node as illustrated in Table 1. The novel genotype INMV 218 has a numerical code of 42331(1.5)18 (Table 2). In order to show the validity of the VNTR typing assay, a tissue homogenate originating from caprine lymph nodes from a different Swiss alpine region (canton of Ticino), which was successfully cultured and identified as a C-type MAP, was identified by VNTR as INMV1 profile. For successful MIRU-VNTR genotyping from tissue homogenates an approximate cut off can be defined for 20 GE of MAP (corresponding to 300 bacteria/analyzed tissue sample) with a calculated 0.1 pg/μl of MAP DNA [approximate C_T_ cut off for IS*900* target: C_T_ = 30 for in-house qPCR; C_T_ = 25 for qPCR by Plain et al. (12)].

**Table 2:** Overview of the MIRU-VNTR genotype observed for the tested goats.

**Number of Tandem repeats at eight MIRU-VNTR loci^a^**								**INMV profile^b^**
292	X3	25	47	3	7^c^	10	32	INMV 218
4	2	3	3	1	1.5	1	8	

a *Eight established MIRU-VNTR loci defined by Thibault et al. (13)*.

b *INMV: INRA Nouzilly MIRU-VNTR*.

c *In addition to one tandem repeat at VNTR locus 7, 12 nucleotides (cgt tcg gcg cgc) were identified as “imperfect” tandem repeat by sequence analysis and were numbered as 1.5 repeats*.

**Figure 3:**
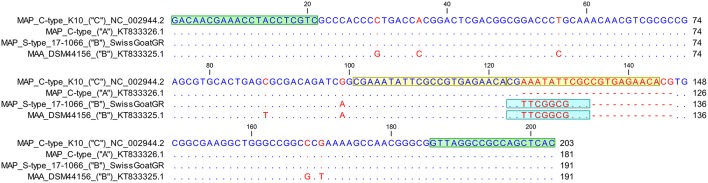
Sequence alignment of VNTR 7 locus of *Mycobacterium avium* subsp. *paratuberculosis* (MAP) C-type reference strain K10 comprising two “perfect” 22 bp long tandem repeats in comparison to a previously published sequence of a MAP C-type isolate with one tandem repeat (GenBank accession number KT833326.1), a S-type III Swiss goat isolate and a *Mycobacterium avium* subsp. *avium* reference strain DSM44156 (GenBank accession number KT833325.1) displaying in addition to a “perfect” tandem repeat a 12 bp “imperfect” repeat. VNTR locus pattern as described previously (24) is indicated in parentheses. VNTR 7 locus pattern “A” stands for one “perfect” tandem repeat, “B” for one “perfect” and one “imperfect” repeat whereas locus pattern “C” represents 2 “perfect” tandem repeats. Primer sequences (2020) are colored in green, whereas light blue highlights the 12 bp long “imperfect” repeat. Yellow represents the sequence of the two “perfect” tandem repeats. Matching residues are marked as blue dots, whereas red indicates non-conserved bases. At position 99 relative to the VNTR 7 forward primer a G→A substitution is detected differentiating MAP C-type from S-type.

The sequence of the VNTR 7 locus displayed a 22 base pair (bp) tandem repeat and a 12 bp long “imperfect” repeat (Figure 3). Therefore, the VNTR code for this locus was determined numerically as 1.5. In addition, one SNP was found; this was at position 99 (G>A) of the amplicon generated by VNTR7 primers and differentiates between C- and S-type (Figure 3) in accordance with previous observations (24).

## Discussion

The present study reports paratuberculosis caused by a new genotype in two goats from a Swiss dairy farm. The pathological features in both animals were consistent with those generally observed with paratuberculosis in small ruminants, comprising a granulomatous inflammation in small intestines (especially jejunum and ileum) and mesenteric lymph nodes (25). However, the number of acid fast bacteria within macrophages in the lesions was moderate to low in both animals. Paucibacillary paratuberculosis has been described in sheep before, where it has been associated with a Th1-type immune response that allows control of the MAP infection via cell-mediated immune response (26). Sheep with the paucibacillary form can recover from the disease or progress to a multibacillary form (27). It remains unclear if the S-type substrain identified in the present study does affect the number of bacteria in the lesions and the morphology/distribution of the lesions, respectively in goat hosts. A recent study in cattle only found weak indication of variable virulence among C-type MAP (28). However, there is evidence of differences in the virulence between C- and S-type strains: while experimental infection of lambs with S-type strains resulted in a persistent granulomatous lymphadenitis in intestine-associated lymph nodes that did not change in intensity over time, C-type strains induced lesions that tended to decrease in severity (29).

MAP was present in highest abundance in the ileum and regional lymph nodes in association with the granulomatous inflammatory infiltrates which harbor the bacilli within lesional macrophages. However, intestinal compartments without overt granulomatous infiltrates, such as colon, caecum and rectum were found to carry bacilli, albeit in lower amounts. The rumen (in one animal) as well as many lymph node samples only harbored low to moderate amounts of MAP. This is in accordance with other studies, in which mycobacteria were occasionally found in extraintestinal and also extranodal tissues (30, 31). The phenotyping of immune cells in the small intestine and mesenteric lymph node, especially the slightly elevated T/B ratio in the nodal tissue, revealed similar results as in an experimental infection in sheep (32) suggesting a Th1-mediated immune response. A contradicting positive MAP result in the aorta despite the lack of morphological changes was interpreted as contamination during sample collection. A granulomatous arteritis which was shown in small and medium sized arteries in goats experimentally infected with MAP could not be detected (25). This observation may underline the more or less paucibacillary form with decreased numbers of excreted bacteria of the involved S-type MAP. Nevertheless, there was a variation in bacterial numbers throughout different compartments of the intestinal tract. The mainly affected parts (jejunum and ileum in goat 1 and ileum in goat 2, respectively) showed moderate numbers of acid fast bacteria whereas other compartments clearly depicted the paucibacillary form. In spite of the varying amount of MAP detected in the different tissues extracted, the shedding of bacteria from clinically healthy animals and consecutively the infection of other animals can be assumed.

The qPCR proposed by Plain et al. (12) displayed an increased detection of MAP around the threshold level with an improved sensitivity, therefore demonstrating a good alternative to a probe-based assay such as the current developed in-house qPCR or a commercially available qPCR kit. However, results with very low numbers of MAP detected in dissected tissue samples below 10 bacteria could possibly also be interpreted as a low level of contamination, which is practically inevitable when dissection of infected animals is performed and thus have to be interpreted very carefully. Anyway, the approach of analyzing *T*_*m*_ of MAP-specific amplification curves seemed to be very promising based on its contribution to an increased specificity and sensitivity. A possible interference with IS*900*-like insertion elements of some mycobacterial species could be detected by a shift of *T*_*m*_. The two sets of goat tissue samples analyzed in this study showed highly reproducible *T*_*m*_ values of 89.6°C ± 0.04 underlining the advantage of using a qPCR assay, which is coupled to a melt curve analysis. The comparison between results obtained from the developed in-house qPCR with those of the qPCR of Plain et al. (12) showed a good correlation with respect to the presence or absence of MAP. The strength of the in-house qPCR is underlying the fact that an internal control is included in the qPCR assay, allowing thereby to monitor potential inhibitory samples, which could possibly remain undetected in a SYBR green based approach. However, as already observed earlier by Acharya et al. (33) no evidence of inhibition was found while performing the qPCR as suggested with fivefold diluted DNA.

The VNTR profile appeared to spread across at least two farms in a regional alpine district of Switzerland (canton of Grisons) since one caprine isolate originating from a farm of a close by neighboring valley (isolated in 2017) was found to have an identical VNTR code. The air-line distance of the two farms is only 6 km and connected by a common alpine pasture, which is used for traditional mountain grazing in the summer. During the common grazing of different goat flocks transmission of the implicated MAP isolate possibly took place.

Ahlstrom et al. and Bryant et al. observed some limitations of VNTR typing by comparing MIRU-VNTR profiles to whole genome sequencing. They suggested that VNTR typing potentially over- and underestimates the relatedness of MAP isolates within a single herd and therefore it is difficult to make any conclusions about transmission dynamics (34, 35). However, considering the geographical proximity of the two farms, the examined strains are likely to have a similar or identical genotype. Since cultivation of S-type MAP strains is very tedious and time-consuming and still ongoing in our laboratory, clarified phylogenetic relationships and genotype-specific virulence characteristics of Swiss S-type MAP strains will only be available in the future. The possibility of detection of implicated VNTR genotypes and subtyping of strains using tissue homogenates without time delay for culturing this fastidious organism seemed to be advantageous when trying to examine an outbreak of a possible emerging pathogen. Rapidly obtained strain genotyping results allow a comparison of internationally described genotypes along with early knowledge of MAP transmission, which could be used for promptly choosing an appropriate eradication strategy. As concluded by Acharya et al. in their work (33) while comparing the sensitive qPCR assay of culture-independent identification of MAP in ovine tissues to histopathological analysis of lesions as well as to bacterial culture, it could be proposed similarly to establish and validate a strategy of routinely testing abattoir intestinal tissue homogenates of small ruminants as well as of cattle including products for human consumption with their suggested highly sensitive qPCR assay coupled to genotyping by VNTR. This procedure could finally lead to an amelioration of the surveillance system, which allows comparing simultaneously identified strains with the internationally established VNTR genotyping databank of MAP. Thereby, a quick recognition of involved strains would rapidly unravel implicated infection chains of MAP.

Worth to mention, VNTR profiles of Swiss S-type MAP strains identified seem to differ only in VNTR loci 292 and X3 when comparing to two other goat and sheep herds from Switzerland (data not shown). Interestingly, the polymorphism of the examined strains found at VNTR locus 7 (1.5 tandem repeats) with the so-called locus pattern “B” involving an “imperfect” tandem repeat as described previously (24) is also found in different S-type MAP isolates of two other goat and two sheep herds from Switzerland (data not shown). This specific polymorphism, in form of an “imperfect” repeat at VNTR locus 7, is more stable than perfect sites (36) and could have a role in adaptation mechanisms of bacteria to changing environmental conditions (37). The downstream *DesA3-1* gene of the VNTR 7 locus encodes a linoeoyl-CoA desaturase (EC 1.14.19.3), which is involved in linoleic acid metabolism and thus has an important function in the cell wall formation of mycobacteria. It is therefore of interest to highlight that a conceivable tandem repeat based regulation mechanism could indicate the evolution of a novel epidemiological lineage of MAP in Switzerland. In conclusion, further investigations on larger number of infected goats originating from the neighborhood of the concerned farms as well as from the entire canton of Grisons and if possible from local wildlife should be performed in order to have a more expanded picture about the epidemiological and pathological pattern of the involved MAP strains. Furthermore, the comparison of both qPCR methods showed a good correlation concerning bacterial load and distribution of bacteria in different tissues according to histochemical examinations. The demonstration of a successful MIRU-VNTR typing using tissue homogenates, nonetheless, enables quick genotyping of involved strains without the necessity of time-demanding MAP cultures. As a consequence, the obtained strain typing results can be used for developing better surveillance and control strategies of MAP and thereby confining further expansion of paratuberculosis in the future.

## Data Availability

All datasets generated for this study are included in the manuscript and/or the Supplementary Files.

## Ethics Statement

In accordance with local legislation, ethical approval was not required and no animal experiments were carried out for this study.

## Author Contributions

SS, FS, RS, and AK designed and coordinated the study. JZ identified and supplied the examined goats. SS and FS performed the experiments and wrote the manuscript. RS and AK reviewed and edited the manuscript. All authors read and approved the final manuscript.

### Conflict of Interest Statement

The authors declare that the research was conducted in the absence of any commercial or financial relationships that could be construed as a potential conflict of interest.
