# Supplementary material for: Morphological and Molecular Characterization of a New Mycobacterium avium Subsp. paratuberculosis S-Type Strain Genotype in Goats
Source: Front Vet Sci. 2019 Jul 31;6:250. doi: 10.3389/fvets.2019.00250 (PMC6684744; doi:10.3389/fvets.2019.00250)
Supplement: Supplementary file 1 [file Image_1.pdf]

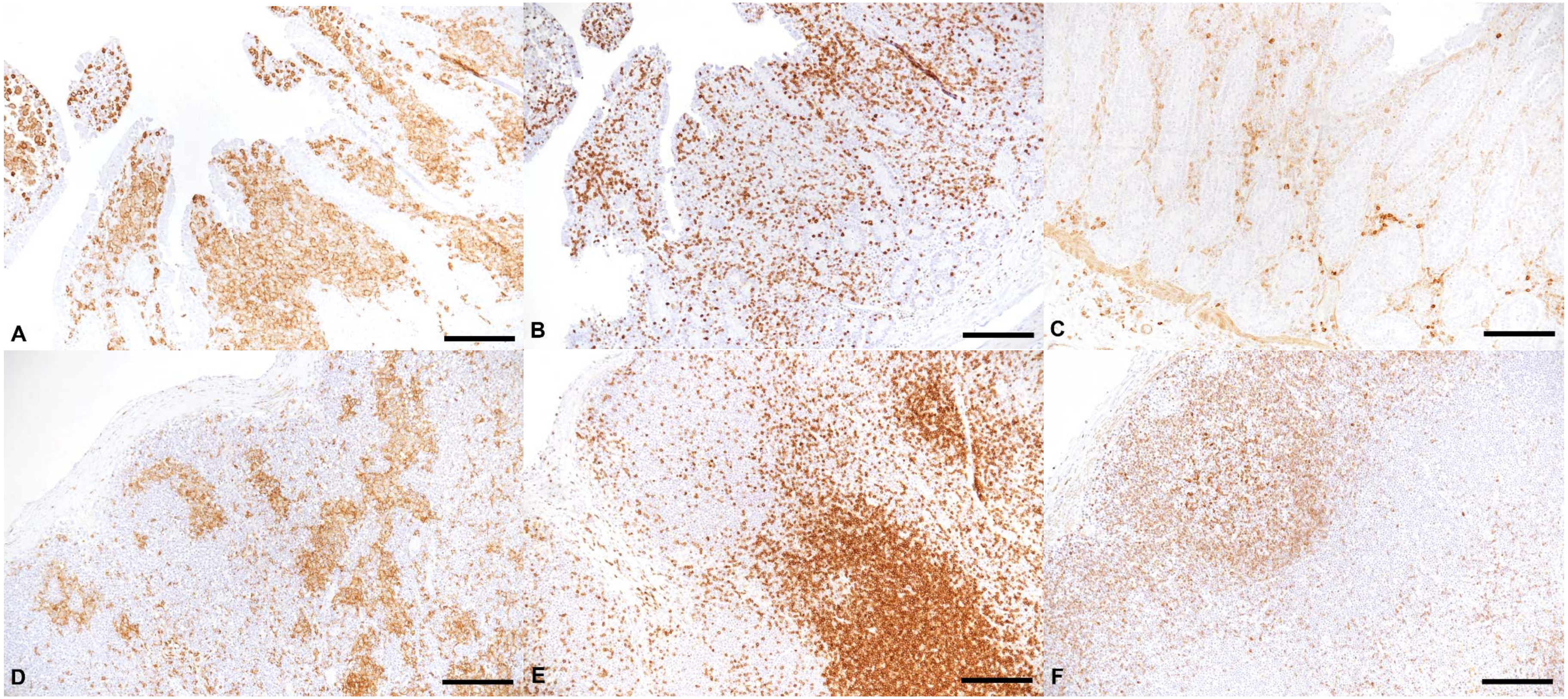

Supplementary Figure S1

Immunohistological detection of macrophages, T and B lymphocytes small intestine and mesenteric lymph node of goats with paratuberculosis (“goat 1”). **(A)** Infiltration of the villous lamina propria with macrophages, Iba1 IHC, bar = 100 µm. **(B)** Diffuse infiltration of the lamina propria with T lymphocytes, CD3 IHC, bar = 100 µm. **(C)** Small number of B lymphocytes in basal areas of lamina propria, CD79a IHC, bar = 100 µm. **(D)** Nodular perifollicular infiltration of epitheloid macrophages in the lymph node, Iba1 IHC, bar = 100 µm. **(E)** Paracortical hyperplasia of T lymphocytes in the mesenteric lymph node, CD3 IHC, bar = 100 µm. **(F)** Follicular hyperplasia of B lymphocytes in the mesenteric lymph node, CD79a IHC, bar = 100 µm. IHC = immunohistochemistry.
